# Keratin 17 is a prognostic and predictive biomarker in pancreatic ductal adenocarcinoma

**DOI:** 10.1093/ajcp/aqae038

**Published:** 2024-04-20

**Authors:** Lyanne A Delgado-Coka, Lucia Roa-Peña, Sruthi Babu, Michael Horowitz, Emanuel F Petricoin, Lynn M Matrisian, Edik M Blais, Natalia Marchenko, Felicia D Allard, Ali Akalin, Wei Jiang, Brent K Larson, Andrew E Hendifar, Vincent J Picozzi, Minsig Choi, Kenneth R Shroyer, Luisa F Escobar-Hoyos

**Affiliations:** Departments of Pathology; Departments of Preventative Medicine, Renaissance School of Medicine, Stony Brook University, Stony Brook, NY, US; Departments of Pathology; Department of Pathology, School of Medicine, Universidad Nacional de Colombia, Bogotá, Colombia; Departments of Pathology; Departments of Pathology; Center for Applied Proteomics and Molecular Medicine, George Mason University, Fairfax, VA, US; Perthera, McLean, VA, US; Scientific and Medical Affairs, Pancreatic Cancer Action Network, Manhattan Beach, CA, US; Perthera, McLean, VA, US; Departments of Pathology; Department of Pathology, University of Arkansas for Medical Sciences, Little Rock, AR, US; Department of Pathology, University of Massachusetts Memorial Medical Center, Worcester, MA, US; Department of Pathology, Anatomy and Cell Biology, Sidney Kimmel Cancer Center Thomas Jefferson University Hospital, Philadelphia, PA, US; Department of Pathology and Laboratory Medicine, Cedars-Sinai Medical Center, Los Angeles, CA, US; Department of Pathology and Laboratory Medicine, Cedars-Sinai Medical Center, Los Angeles, CA, US; Virginia Mason Medical Center, Seattle, WA, US; Departments of Pathology; Departments of Pathology; Departments of Pathology; Departments of Therapeutic Radiology; Departments of Molecular Biophysics and Biochemistry; Department of Medicine, Division of Oncology, Yale University, New Haven, CT, US

**Keywords:** pancreatic ductal adenocarcinoma, chemotherapies, predictive biomarkers, immunohistochemistry

## Abstract

**Objectives:**

To determine the role of keratin 17 (K17) as a predictive biomarker for response to chemotherapy by defining thresholds of K17 expression based on immunohistochemical tests that could be used to optimize therapeutic intervention for patients with pancreatic ductal adenocarcinoma (PDAC).

**Methods:**

We profiled K17 expression, a hallmark of the basal molecular subtype of PDAC, by immunohistochemistry in 2 cohorts of formalin-fixed, paraffin-embedded PDACs (n = 305). We determined a K17 threshold of expression to optimize prognostic stratification according to the lowest Akaike information criterion and explored the potential relationship between K17 and chemoresistance by multivariate predictive analyses.

**Results:**

Patients with advanced-stage, low K17 PDACs treated using 5-fluorouracil (5-FU)–based chemotherapeutic regimens had 3-fold longer survival than corresponding cases treated with gemcitabine-based chemotherapy. By contrast, PDACs with high K17 did not respond to either regimen. The predictive value of K17 was independent of tumor mutation status and other clinicopathologic variables.

**Conclusions:**

The detection of K17 in 10% or greater of PDAC cells identified patients with shortest survival. Among patients with low K17 PDACs, 5-FU–based treatment was more likely than gemcitabine-based therapies to extend survival.

**KEY POINTS**

• There is an urgent need to establish readily deployable biomarker-based assays to guide optimal therapeutic interventions and to limit toxic side effects.
• Incorporating keratin 17 (K17) immunohistochemistry (IHC) testing as a predictive marker test will yield rapid results to inform survival and the best chemotherapy based on the tumor’s K17 expression profile.
• 5-Fluorouracil rather than gemcitabine-based therapies is more likely to extend survival for patients’ tumors expressing low rather than high K17, and thus K17 IHC could guide therapeutic interventions for pancreatic ductal adenocarcinoma.

## INTRODUCTION

Although the 5-year survival for patients with pancreatic ductal adenocarcinoma (PDAC) has marginally improved from 5% to 12% over the past 4 decades, PDAC is projected to become the second leading cause of cancer death in the United States by 2030.^1^ Multiagent chemotherapeutic combinations, including gemcitabine plus nab-paclitaxel, and 5-fluorouracil (5-FU), folinic acid, irinotecan, and oxaliplatin (FOLFIRINOX) are the standard first-line choices, although for subgroups of patients, these treatments only provide limited efficacy with significant toxicity.^2,3^ Thus, the development of readily deployable and rapid biomarker-based assays to guide optimal chemotherapeutic interventions based on specific molecular/biologic properties of each individual patient holds great promise to drive major advances in treatment and survival.

It was previously reported that keratin 17 (K17) expression, detected at either the level of messenger RNA (mRNA) or protein by immunohistochemistry (IHC), is as accurate as molecular subtyping to identify subgroups of patients with PDAC with the shortest survival.^4^ Remarkably, multiple teams of investigators independently identified K17 mRNA expression as a component of molecular signatures that identify the most aggressive subtypes of PDAC, and K17 is now widely accepted as a defining marker of the basal molecular subtype.^5-7^ Thus, we developed a K17 IHC test to define the molecular subtype, which has been validated in resected specimens for low-stage disease and cell blocks of needle biopsy specimens for nonresectable cases, which account for 85% of PDAC cases.^8^

While the standard of care for resectable PDAC is surgical resection followed by 6 months of adjuvant chemotherapy, studies have emerged over the past decade to identify biomarker-based approaches to provide tailored care for patients with PDAC. Subsequent clinical trials have consistently used both 5-FU and gemcitabine as backbone chemotherapy treatments; however, treatment-related toxicity, impact on quality of life, and limited survival gains mean that selecting appropriate patients for each intervention is challenging and highlights the need of predictive biomarkers of response to chemotherapy that could potentially aid patient management. To the best of our knowledge, no studies have been published correlating a surrogate marker of the basal PDAC subtype biomarker with response to chemotherapy.

In the current study, we determined the predictive value of K17 testing for both chemotherapeutic approaches to treat PDACs in a cohort of 305 patients with PDAC, including those with localized and metastatic disease who underwent adjuvant chemotherapy. Collectively, our results indicate that K17 expression is a robust, predictive biomarker for 5-FU vs gemcitabine-based chemotherapies in advanced-stage PDACs.

## METHODS

### Patient Demographics

**FIGURE 1:**
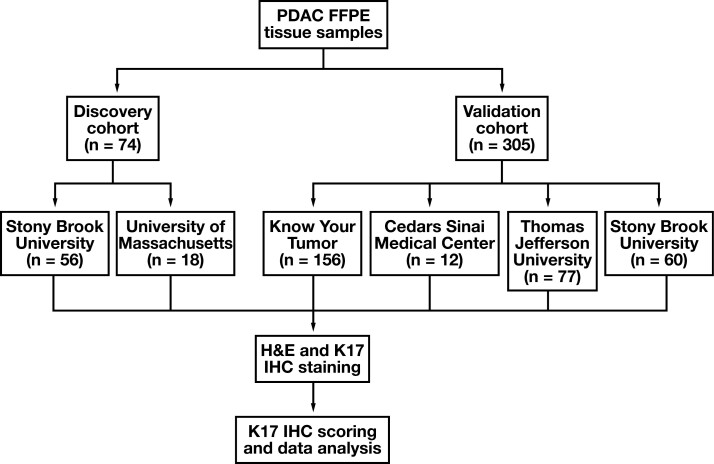
Flowchart, analysis of keratin 17 in 2 differential cohorts of PDAC. FFPE, formalin-fixed, paraffin-embedded; IHC, immunohistochemistry; K17, keratin 17; PDAC, pancreatic ductal adenocarcinoma.

The discovery cohort^4^ comprised archival formalin-fixed, paraffin-embedded (FFPE) tumors collected between 2008 and 2012 from patients with no history of neoadjuvant treatment at Stony Brook University Hospital and the University of Massachusetts (n = 74) who mainly received gemcitabine as adjuvant therapy FIGURE 1.

The validation cohort included 305 PDAC tissue blocks from 2013 to 2019 that were collected from the Pancreatic Cancer Action Network Know Your Tumor (KYT) program (n = 156), a national cohort coordinated by Perthera, Stony Brook Medicine (SBU) (n = 60), Cedars-Sinai Medical Center (n = 12), and Thomas Jefferson University (n = 77). Most (87%) of these cases were from primary resections, 4% were biopsy specimens, and 9% were biopsy specimens from metastatic sites. Seventy-five percent of these cases were from the KYT program and processed at Perthera for molecular profiling to identify treatment options based on actionable biomarkers. Moreover, 21% of KYT cases received neoadjuvant treatment and 78% of KYT patients (n = 121) received adjuvant chemotherapy and were followed longitudinally to track physician treatment choices and survival outcomes. The remaining 25% of the entire validation cohort was composed from samples selected from SBU and other collaborating sites that also underwent adjuvant treatment.

### Selection Criteria

H&E-stained sections from all tumor blocks were reviewed from each case to identify the tissue block to be included in the study, based on its representation of the greatest area of viable tumor. A pilot study was performed to assess for spatial heterogeneity of K17 expression and to evaluate the reproducibility of pathologist readout. Ten PDAC cases were randomly selected from the SBU archival collection, and 5 FFPE blocks were stained and scored from each case. Two pathologists (K.R.S. and L.R.-P.) independently scored each tissue block for the percentage of tumor cells that expressed K17. Two tissue blocks were excluded because tissue was lost from the slide during antigen retrieval and/or subsequent processing for IHC. Overall, the percentage of tumor cells that stained for K17 was consistent (differences ≤15%) in 46 of 48 tissue blocks (*r* = 0.9550; 95% CI, 0.9211-0.9745; *P* < .0001) (Supplementary Table 1; all supplementary material is available at *American Journal of Clinical Pathology* online). Therefore, 1 block was selected per patient, and selection criteria of tumor specimens were followed, as previously described.^9^ Briefly, patients were stratified based on the American Joint Committee on Cancer (AJCC) eighth edition staging criteria, as recorded in the original surgical pathology diagnostic reports. Tumor stages T1 to T3 were defined according to tumor size (T1 ≤ 2 cm; 2 cm ≥ T2 ≤ 4 cm; T3 > 4 cm) of tumor limited to the pancreas (T1-T2) vs tumors that extended beyond the pancreas (T3-T4); lymph node (LN) status was recorded as no regional metastases (N0), 1 to 3 regional metastases (N1), or more than 3 LN regional metastases (N2). Histologic grades were grouped into well and moderately differentiated (G1-G2) vs poorly differentiated (G3). Survival and adjuvant therapy data were obtained from the respective institution’s registry. Tumor stage was assigned based on AJCC criteria,^10^ and histopathologic grade was based on World Health Organization criteria.^11^ All analyses were performed in accordance with these criteria.

### Ethics Statement

All members of our research team completed Collaborative Institutional Training Initiative human subjects and medical ethics training, and all studies were performed in accordance with guidelines and regulations of the Stony Brook Medicine Institutional Review Board (IRB) protocol 94,651. Patient consent was waived by the IRB agreements for each participating site because the study was restricted to the analysis of deidentified remnant waste surgical pathology specimens. Tissue slides were deidentified, and multiple security measures, including password protection and storage of the password key on a computer without network access, were used to ensure that no patient identifiers could be accessed.

### K17 IHC Staining

Histologic sections of PDAC from 2 independent patient cohorts (discovery and validation cohorts) were processed for manual K17 IHC TABLE 1. An indirect immunoperoxidase method was used to identify the presence of K17, as previously described.^12^ Briefly, after incubation at 60°C, sections were deparaffinized in xylene and rehydrated in graded alcohols. Antigen retrieval was performed in citrate buffer at 120°C for 10 minutes in a decloaking chamber. Of note, less stringent conditions for heat-induced epitope retrieval at 100°C produced lower scores for K17, in terms of both the fraction of positive cells and stain intensity. Then, endogenous peroxidase was blocked by 3% hydrogen peroxide, and sections were incubated overnight at 4°C with mouse monoclonal anti-human K17 antibody (KDx). After application of the primary antibody, biotinylated horse secondary antibodies (R.T.U. Vectastain ABC kit; Vector Laboratories) were added. Development was performed with 3,3ʹ diaminobenzidine (Dako), and counterstaining was done with hematoxylin. Negative controls were included in all runs using an equivalent concentration of a subclass-matched immunoglobulin, as previously described.^9^

**TABLE 1:** Patient Cohort Demographics^a^

Characteristic	Discovery cohort	Validation cohort
Total No. of cases included	74	305
Follow-up, mean ± SD, mo	17.8 ± 15.2	22.2 ± 18.5
Age at diagnosis, mean ± SD, y	65.5 ± 9.9	64.5 ± 9.7
Sex, No. (%)		
Female	38 (51)	141 (46)
Male	36 (49)	164 (54)
Histologic grade (G), No. (%)		
G1 + G2, well and moderately differentiated	42 (57)	205 (67)
G3, poorly differentiated	32 (43)	88 (29)
GX, cannot be assessed	—	12 (4)
LN status, No. (%)		
LN negative	26 (35)	69 (23)
LN positive	48 (65)	236 (77)
AJCC eighth edition pathological stage, No. (%)		
Early stage (I-IIB)	72 (97)	230 (75)
Advanced stage (III-IV)	2 (3)	75 (25)
Specimen type, No. (%)		
Biopsy	—	12 (4)
Resection	74 (100)	293 (96)
Tumor, No. (%)		
Primary tumor	74 (100)	278 (91)
Metastasis	—	27 (9)
Adjuvant therapy in IIB-IV stage, No. (%)		
Gemcitabine-based chemotherapy	15 (11)	127 (89)
Gemcitabine only	12 (23)	40 (77)
5-FU–based chemotherapy	3 (4)	83 (96)
FOLFIRINOX	3 (5)	59 (95)
Not adjuvant treatment	3 (7)	41 (93)
Radiotherapy	–	33 (11)

AJCC, American Joint Committee on Cancer; K17, keratin 17; LN, lymph node; PDAC, pancreatic ductal adenocarcinoma; 5-FU, 5-fluorouracil.

^a^Gemcitabine-based therapy includes the following: gemcitabine, gemcitabine and nab-paclitaxel, or any other chemotherapy combination that includes gemcitabine. 5-FU–based therapy includes the following: FOLFIRINOX, FOLFIRI, FOLFOX, or any other chemotherapy combination that includes 5-fluorouracil. Radiotherapy includes all forms of radiation in combination with or without other chemotherapeutic agents.

### K17 IHC Scoring

The K17 staining intensity was independently scored, blinded to corresponding clinical data, by 2 pathologists (K.R.S. and L.R.-P.) based on the percentage of strong (2+) stained tumor cells within a single representative histologic section from each case (PathSQ score^9^). Scoring discrepancies between pathologists were resolved by consensus after a joint review.

### Mutation Profile Assessment

Data on cancer-related mutations were obtained from Perthera. DNA sequencing was performed as previously reported by Pishvaian et al.^13^ Shortly, tumor samples from patients with biopsy-confirmed PDAC were sent for next-generation sequencing (NGS) analysis of cancer-related mutations. Patient tumor genomic profiles were then reviewed, and pathogenic mutations were confirmed by Perthera’s molecular tumor board participants on a case-by-case basis.^13^

### Statistical Analysis

Patients were categorized based on their clinicopathologic features into meaningful groups, and the χ^2^ test and Fisher exact test were used to analyze associations between 2 categorical variables. The best cutoff point to stratify patient survival was chosen according to the lowest Akaike information criterion (AIC) from a Cox proportional hazard regression model to determine low vs high K17 protein expression. Survival curves were plotted using the Kaplan-Meier method, hazard ratios (HRs) were calculated using Cox proportional hazard regressions, and survival rates were compared by means of the log-rank test. Univariate and multivariate analyses compared survival based on K17 expression score with clinical and various pathologic factors by Cox proportional hazards regression. Overall survival (OS) was calculated from the initial diagnosis of disease until death, and progression-free survival was defined as the period from start of treatment to death for any reason or to recurrence of disease. Statistical significance was set at *P* ≤ .05, and analysis was done using SAS 9.4 (SAS Institute) and GraphPad Prism 7 (Graph Pad Software). All *P* values were calculated using a 2-sided test.

## RESULTS

### Definition of K17 Status in Patient Cohorts

**FIGURE 2:**
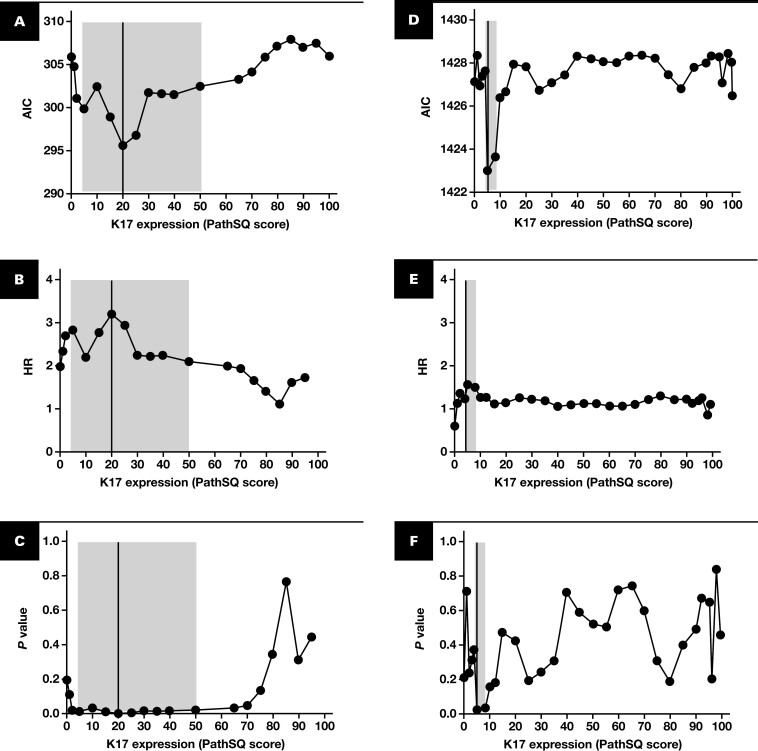

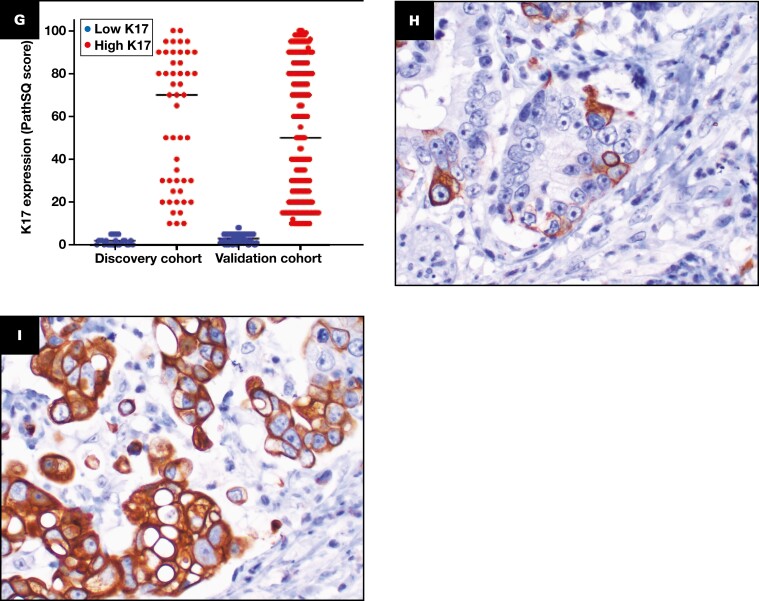
Identification of optimal cutoff value for K17 on both discovery (**A-C**) and validation (**D-F**) cohorts, respectively. **A**, Akaike information criterion (AIC). *P* value (**B**) and HR values (**C**) plotted as a function of K17 expression PathSQ score in the discovery cohort and in the validation cohort (**D-F**). Gray shaded areas in **A-F** represent areas where *P* value was significant. **G**, Boxplot depicting K17 expression levels in the entire cohort (n = 379). Representative images of moderately differentiated PDACs with low K17 (**H**) and high K17 (**I**) expression. AIC, Akaike information criterion; HR, hazard ratio; K17, keratin 17; PDAC, pancreatic ductal adenocarcinoma.

The threshold providing maximal stratification of survival differences based on K17 detection was determined in the discovery cohort. We used the lowest AIC, with the maximal and significant Cox proportional hazard regression model. The threshold with the most significant (log-rank test) differences in the OS between the high K17 and low K17 groups was 20% (expression of K17 in 20% of tumor cells), but significant differences were found for scores ranging from 5% to 70% FIGURE 2A-2C. In the validation cohort, we found significant differences in scores ranging from 5% to 10% FIGURE 2D-2F. The exclusion of biopsy specimens (n = 12) and/or metastatic sites (n = 27) did not affect the results. Thus, we chose 10% as the cutoff point that best stratified both cohorts combined and improved the clinical utility of IHC findings. Therefore, patients with a strong expression of K17 detected in 10% or more of malignant cells were classified as having high K17 PDACs, and those with a strong stain in less than 10% of tumor cells were classified as having low K17 PDACs. Based on this threshold, the low K17 group comprised 34% of the cases, whereas the high K17 group included 66% in the discovery cohort. In the validation cohort, the low K17 group comprised 25% of cases and the high K17 group included 75% of cases FIGURE 2G-2I.

### K17 Expression Is Independent of Driver Mutations in PDAC

To determine if K17 expression was associated with the mutation status of the tumors, we conducted analyses using the KYT cohort, where 146 of cases were sequenced for driver mutations using NGS. As expected, *KRAS* (94%), *p53* (71%), *CDKN2A* (41%), and *SMAD4* (25%) mutations were the top 4 most mutated genes in PDAC (Supplementary Figure 1A). We found that K17 expression was not increased based on any given mutation (Supplementary Figure 1B-1E). These results support previous findings that K17 expression, as the expression of other genes that define the molecular subtypes, is not influenced by mutational status of the patient.^5-7^

### High K17 Status Is Associated With Worst Patient Outcome

**FIGURE 3:**
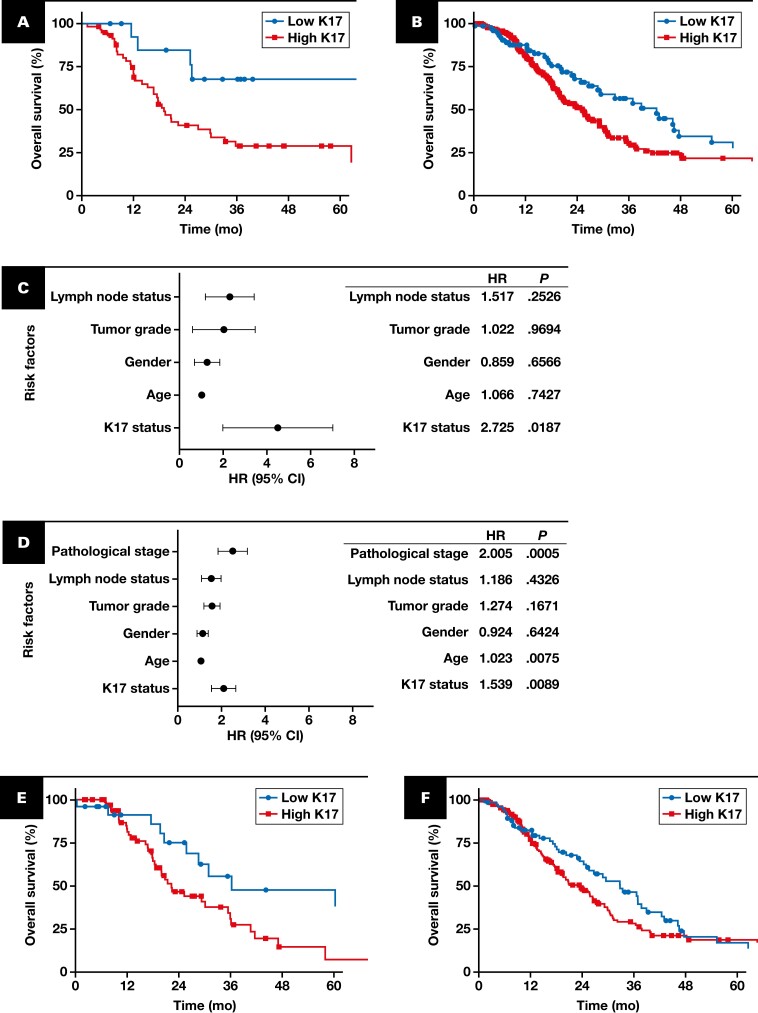

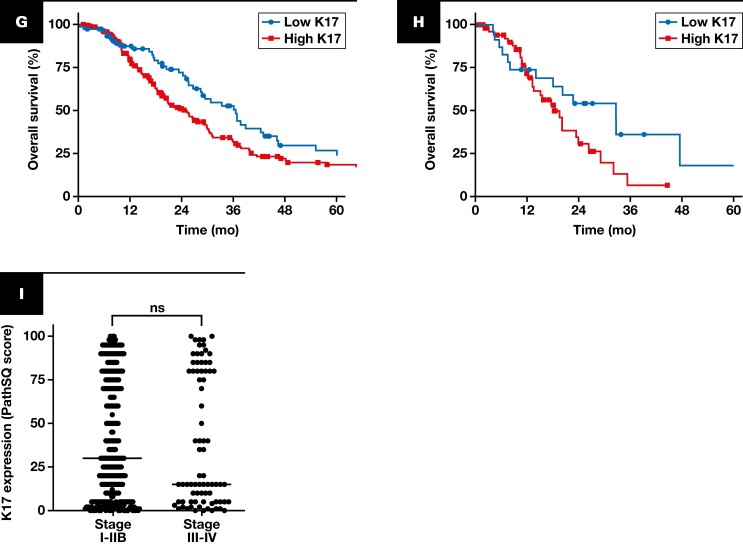
Validation of K17 as an independent negative prognostic biomarker of PDAC. Kaplan-Meier curves for the overall survival analysis of K17 from PDAC cases in the discovery (**A**) and validation (**B**) cohorts. **A**, Low K17 (n = 15 [25%]; median = 19 months) and high K17 (n = 59 [75%]; median = 27 months). HR = 3.217 (95% CI, 1.604-6.450); *P* = .0177. **B**, Low K17 (n = 76 [25%]; median = 42 months); high K17 (n = 229 [75%]; median = 25 months). HR = 1.511 (95% CI, 1.065-2.124); *P* = .0338. Forest plots showing the multivariate analysis factors in the discovery (**C**) and validation (**D**) cohorts. Kaplan-Meier curves depict the overall survival, which integrate K17 status and the lymph node status for the combined discovery (**E**) and validation (**F**) cohorts. **E**, Lymph node–negative cases. Low K17 (n = 25 [26%]; median = 36 months); high K17 (n = 70 [74%]; median = 22 months). HR = 2.119 (95% CI, 1.174-3.823); *P* =.0258. **F**, Lymph node–positive cases. Low K17 (n = 76 [27%]; median = 33 months); high K17 (n = 208 [73%]; median = 23 months). HR = 1.272 (95% CI, 0.9163-1.765); *P* = .1659. Kaplan-Meier curves depict the overall survival, which integrate K17 status and pathological stage for the combined discovery (**G**) and validation (**H**) cohorts. **G**, Early-stage (I-IIB) cases. Low K17 (n = 77 [26%]; median = 36.6 months); high K17 (n = 225 [74%]; median = 25.1 months). HR = 1.441 (95% CI, 1.042-1.997); *P* =.0392. **H**, Advanced-stage (III-IV) cases. Low K17 (n = 23 [30%]; median = 33 months); high K17 (n = 54 [70%]; median = 18 months). HR = 1.772 (95% CI, 0.941-3.151); *P* =.081. *P* values were calculated with the log-rank test. Due to low number of advanced-stage patients, a link between K17 expression and pathologic stage could not be performed within the discovery cohort. The HRs and *P* values are shown for all panels. **I**, Graph showing expression of K17 immunohistochemistry for each case within the same tumor stage category. PathSQ score ranges from 0% to 100% in both categories. *P* value was calculated using the Mann-Whitney test. HR, hazard ratio; K17, keratin 17; ns, not significant; PDAC, pancreatic ductal adenocarcinoma.

By Kaplan-Meier analysis, patients of the discovery cohort with high K17 expression in PDAC had significantly shorter OS (median, 19 months; HR, 3.217; *P =* .0177) than those in the low K17 expression group (median, 27 months) FIGURE 3A. To validate the prognostic value of K17 in a more clinically diverse group of PDACs, we applied the same threshold (10%) in our validation cohort and confirmed that high levels of K17 remained a negative prognostic biomarker, with a median survival of 25 months for high K17 cases (HR, 1.511; *P =* .0338) compared with those in the low K17 expression group (median, 42 months) FIGURE 3B. To evaluate whether the K17 status is independent of other clinicopathologic features, univariate and multivariate analyses using Cox proportional hazards regression of individual risk factors were performed. Importantly, K17 status was independent of other clinicopathologic features by univariate (Supplementary Figure 2) and multivariate FIGURE 3C, 3D analyses using Cox proportional hazards regression. Furthermore, these differences in survival were found within cases with negative LN status FIGURE 3E and within cases with early stage tumors FIGURE 3G. While not significant, high K17 within positive lymph node status and advanced-stage cases tended to have shorter survival than corresponding low K17 counterparts FIGURE 3F, 3H-3I. In summary, results from the discovery and validation cohorts show that high K17, defined based on detection in 10% or more of tumor cells, is an independent prognostic biomarker for patients with early-stage PDAC.

### K17 Predicts Response to Chemotherapy in Advanced-Stage PDAC

**FIGURE 4:**
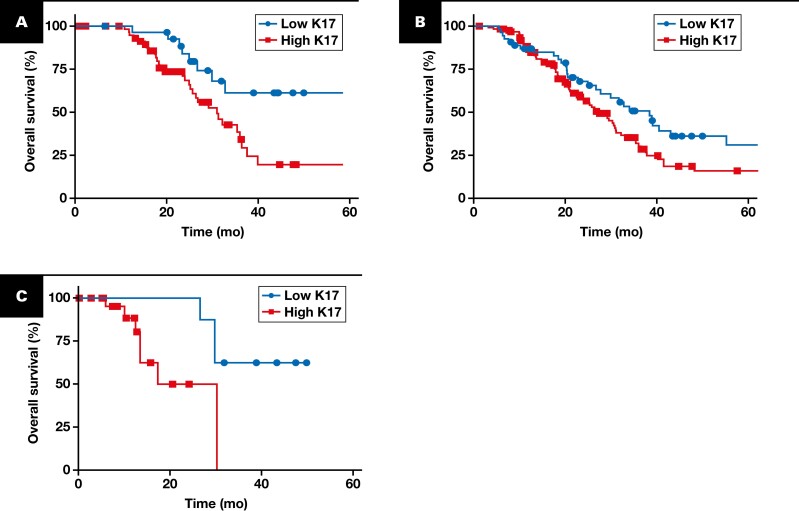
High K17 expression is correlated with shorter survival in patients treated with gemcitabine-based, 5-FU–based therapies or radiotherapy. Kaplan-Meier curves for the overall survival analysis of K17 from PDAC cases of all stages in both cohorts combined. **A**, Overall survival in patients with PDAC treated with 5-FU–based therapies. Low K17 (n = 29 [32%]; median = 69.5 months); high K17 (n = 61 [68%]; median = 31 months). HR = 2.256 (95% CI, 1.220-4.172); *P* = .0118. **B**, Overall survival in patients with PDAC treated with gemcitabine-based therapies. Low K17 (n = 54 [30%]; median = 38.4 months); high K17 (n = 126 [70%]; median = 27.2 months). HR = 1.549 (95% CI, 1.050-2.284); *P* = .0309. **C**, Overall survival in patients with PDAC treated with radiotherapy. Low K17 (n = 8 [24%]; median = 38 months); high K17 (n = 25 [76%]; median = 28 months). HR = 3.678 (95% CI, 1.031-13.12); *P* = .0214. *P* values were calculated using the log-rank test. Gemcitabine-based therapies include the following: gemcitabine, gemcitabine and nab-paclitaxel, or any other chemotherapy combination that includes gemcitabine; 5-FU–based therapies include the following: FOLFIRINOX, FOLFIRI, FOLFOX, or any other chemotherapy combination that includes 5-fluorouracil. Radiotherapy includes all forms of radiation in combination with or without other chemotherapeutic agents. HR, hazard ratio; K17, keratin 17; PDAC, pancreatic ductal adenocarcinoma.

We next analyzed K17 expression in the context of survival after adjuvant chemotherapy. High K17 status was associated with shorter OS of patients treated with either 5-FU–based therapies (FOLFIRINOX, FOLFIRI, FOLFOX) (median, 31 months; HR, 2.256; *P* = .0118) or gemcitabine-based therapies (gemcitabine, gemcitabine and nab-paclitaxel) (median, 27.2 months; HR, 1.549; *P* = .0309) compared to low K17-expressing cases FIGURE 4A, 4B. Although it is widely known that age and other comorbidities affect therapeutic benefit, the lack of comorbidity information prevents us from analyzing these data; the mean age of patients treated with 5-FU–based therapies was 60 years, whereas the mean age of patients treated with gemcitabine-based therapies was 66 years. Considering this limitation, the following analysis was done in a subset of patients who did not receive neoadjuvant treatment, adjusting the analysis for other clinicopathologic features that can also affect chemotherapeutic response, including LN status and tumor stage. Additionally, we observed that patients with high K17-expressing PDACs treated by radiation (with or without chemotherapy) had a shorter survival (median, 28 months; HR, 3.678; *P* = .0214) when compared to low K17-expressing PDACs FIGURE 4C.

**FIGURE 5:**
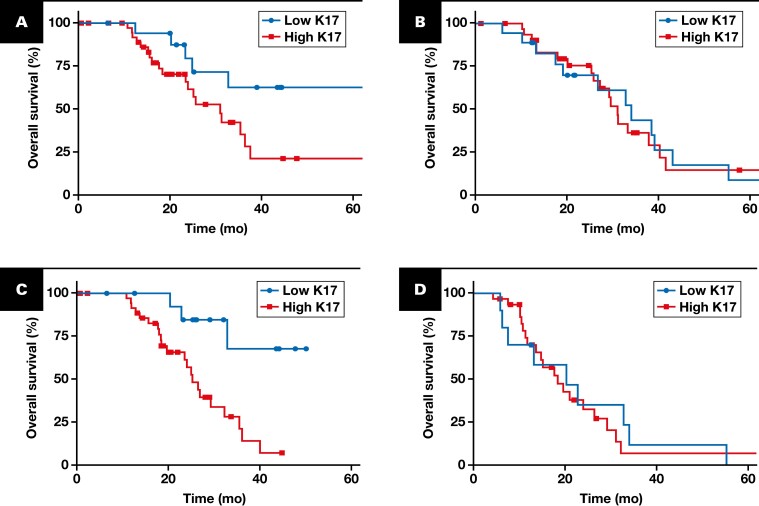
K17 immunohistochemistry prediction of overall survival in lymph node–positive and advanced PDAC according to receipt of chemotherapy. Kaplan-Meier curve for overall survival in patients with lymph node–positive PDAC receiving adjuvant FOLFIRINOX (**A**) and patients receiving gemcitabine (**B**). **A**, Low K17 (n = 18 [31%]; median = 69.5 months); high K17 (n = 40 [69%]; median = 31 months). HR = 2.141 (95% CI, 1.006-4.558); *P* = .0431. **B**, Low K17 (n = 18 [35%]; median = 34 months); high K17 (n = 34 [65%]; median = 31 months). HR = 1.078 (95% CI, 0.5346-2.172); *P* = .8295. Kaplan-Meier curve for overall survival in patients with advanced-stage PDAC receiving adjuvant 5-FU–based chemotherapies (**C**) and in patients receiving adjuvant gemcitabine-based chemotherapies (**D**). **C**, Low K17 (n = 15 [29%]; median = 29 months); high K17 (n = 37 [71%]; median = 25 months). HR = 5.016 (95% CI, 2.258-11.14); *P* = .003. **D**, Low K17 (n = 10 [24%]; median = 20.3 months); high K17 (n = 31 [76%]; median = 18.4 months). HR = 1.117 (95% CI, 0.5201-2.401); *P* = .7684. *P* values were calculated using the log-rank test. Gemcitabine-based therapies include the following: gemcitabine, gemcitabine and nab-paclitaxel, or any other chemotherapy combination that includes gemcitabine. The 5-FU–based therapies include the following: FOLFIRINOX, FOLFIRI, FOLFOX, or any other chemotherapy combination that includes 5-fluorouracil. HR, hazard ratio; K17, keratin 17; PDAC, pancreatic ductal adenocarcinoma.

**FIGURE 6:**
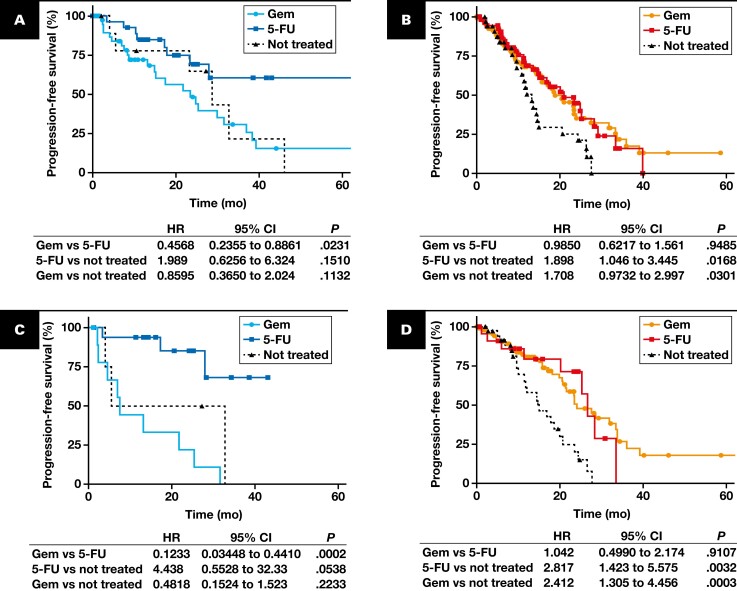

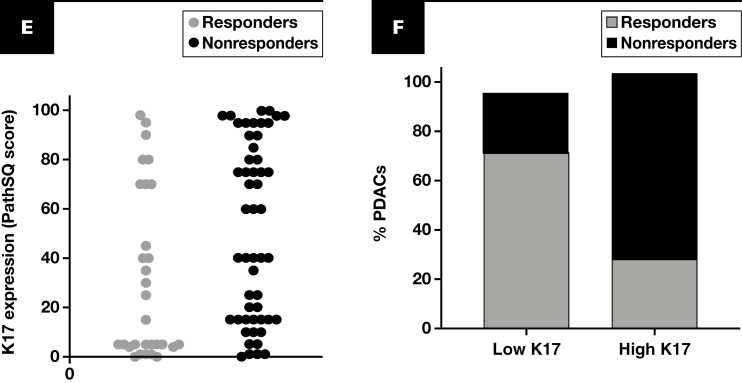
High K17 expression predicts poor therapeutic response to 5-FU–based and gemcitabine (Gem)–based therapies in advanced-stage PDAC. **A-D**, Kaplan-Meier curve for progression-free survival in patients with lymph node–positive PDAC treated with gemcitabine-based or 5-FU–based therapies with low K17 expression (**A**) and high K17 expression (**B**). **A**, Gemcitabine (n = 41 [51%]; median = 23.5 months); 5-FU (n = 29 [36%]; median = 67.2 months); not treated (n = 10 [13%]; median = 28.7 months). **B**, Gemcitabine (n = 100 [51%]; median = 18.9 months); 5-FU (n = 61 [31%]; median = 21.0 months); not treated (n = 34 [17%]; median = 13.3 months). *P* values were calculated using the log-rank test. Kaplan-Meier curve for progression-free survival in patients with advanced-stage (III-IV) PDAC treated with gemcitabine-based or 5-FU–based therapies with low K17 expression (**C**) and high K17 expression (**D**). **C**, Gemcitabine (n = 10 [34%]; median = 7.5 months); 5-FU (n = 15 [52%]; median = 21.3 months); not treated (n = 4 [14%]; median = 19.1 months). **D**, Gemcitabine (n = 31 [40%]; median = 9.9 months); 5-FU (n = 35 [45%]; median = 15.2 months); not treated (n = 12 [15%]; median = 11.7 months). **E**, Comparison of K17 expression levels in responders and nonresponders with lymph node–positive PDACs treated with gemcitabine- or 5-FU–based therapies using RECIST v1.1 criteria. *P* value was calculated using the Mann-Whitney test. **F**, Percentage of patients’ response based on RECIST v1.1 in low and high K17-expressing lymph node–positive PDACs. Gemcitabine-based therapies include the following: gemcitabine, gemcitabine and nab-paclitaxel, or any other chemotherapy combination that includes gemcitabine; 5-FU–based therapies include the following: FOLFIRINOX, FOLFIRI, FOLFOX, or any other chemotherapy combination that includes 5-fluorouracil. *P* value was calculated with the Welch test. HR, hazard ratio; K17, keratin 17; PDAC, pancreatic ductal adenocarcinoma; RECIST, Response Evaluation Criteria in Solid Tumors.

To evaluate the feasibility and clinical utility of K17 to guide chemotherapy selection in LN-positive PDACs, we compared the OS of patients who were treated with FOLFIRINOX, gemcitabine, gemcitabine-based, or 5-FU–based chemotherapies using the cutoff threshold of 10%, as defined above. Our analysis revealed that patients with PDAC with LN-positive status and low K17 status who were treated with FOLFIRINOX had an OS of 69.5 months compared with 31 months for high K17 status patients (*P* = .0431) FIGURE 5A. A similar trend was observed when studying a more broad chemotherapy group that included 5-FU–based chemotherapies in which low K17 patients had a longer survival (median, 29 months; HR, 5.016; *P* = .0030) than their high K17 counterparts FIGURE 5C. Interestingly, there were no benefits seen when we compared the survival of patients treated with either gemcitabine (HR, 1.078; *P* = .8295) or gemcitabine-based chemotherapies (HR, 1.117; *P* = .7684) in low and high K17 patients FIGURE 5B, 5D. Next, we analyzed the progression-free survival (PFS) using the cutoff threshold of 10%, as defined above, and compared each treatment head to head within low K17 status and high K17 groups. The PFS of low K17 status patients who were not treated or treated with either gemcitabine- or 5-FU–based chemotherapies was analyzed. Similarly, we compared the same subgroup of patients within either therapy who had high K17 status. Our analysis revealed that patients with PDAC with LN-positive status and low K17 status who were treated with 5-FU–based chemotherapies had a PFS of 67.2 months compared with 23.5 months for low K17 status patients treated with gemcitabine-based chemotherapies (*P* = .0231) FIGURE 6A. In contrast, we did not find any significant difference in treatment response within the high K17 status patients (*P* = .9485) FIGURE 6B. Similar results were observed when we refined the therapies to be just gemcitabine or FOLFIRINOX (Supplementary Figure 3A, 3B). There were no significant differences in survival between treated and not treated patients with low K17 expression while significant differences were seen in patients within the high K17 expression group FIGURE 6A, 6B (Supplementary Figure 3A, 3B).

We then evaluated the clinical utility of K17 to guide chemotherapy selection based on tumor stage. By Kaplan-Meier analysis, the median survival of patients with advanced-stage PDAC and low K17 treated with 5-FU–based therapies was 21.3 months compared with 7.5 months for patients treated with gemcitabine-based therapies (HR, 0.1233; *P* = .0002) FIGURE 6C. Remarkably, there was no PFS difference in patients with advanced-stage (III-IV) and high K17 PDACs, but patients treated with either regimen did have a significantly improved PFS compared with patients not treated FIGURE 6D. Similarly, there was not a survival difference between patients with early-stage PDACs who expressed high K17 treated with either gemcitabine-based or 5-FU–based therapies (Supplementary Figure 4A, 4B). To test if other thresholds of K17 status had also predictive value, we ran multiple tests and found that the same significant correlations with response to chemotherapy were seen at multiple K17 thresholds (Supplementary Figure 5). Overall, our findings suggest that K17 has both predictive and prognostic implications based on criteria proposed by Ballman,^12^ as we observed a correlation with outcome regardless of treatment and also observed differential treatment effects between the low and high K17-expressing patients. This suggests that patients with systemic disease and low K17 expression in the tumors have better survival when treated with 5-FU–based chemotherapy, but patients with high K17-expressing PDACs do not elicit survival differences based on either chemotherapy treatment.

Last, to gain more insights into the association between K17 expression and response to treatment, we used Response Evaluation Criteria in Solid Tumors version 1.1 data available for a subset of patients (n = 82). Patients were classified as responders, including patients with confirmed complete response, partial response, or stable disease at the time of last follow-up, or nonresponders, including patients with progressive disease at the time of last follow-up. We found that K17 expression within the responder group was 4 times significantly lower (10%, n = 30) than that in patients within the nonresponder group (50%, n = 52) (*P* < .005) FIGURE 6E. Within the responder group, 24% of patients exhibited high K17 status and 72% exhibited low K17 status FIGURE 6F. These results suggest that K17 testing might be leveraged to better inform selection of adjuvant chemotherapy in advanced-stage PDACs.

## DISCUSSION

One of the most clinically important hallmarks of PDAC is its remarkable therapeutic resistance, which has been attributed to several key genetic, metabolic, and immune features of the heterogeneous and hostile microenvironment of these tumors.^14^ Although the 2 most common used adjuvant chemotherapeutic regimens, FOLFIRINOX and gemcitabine, provide significant survival benefit in patients with PDAC, there is lack of availability of predictive biomarkers that can guide personalized therapeutic decision-making. Our results suggest that patients with advanced disease and low K17 benefit from 5-FU–based therapies compared with gemcitabine-based chemotherapies, whereas patients with advanced disease with expression of K17 in 10% or more of tumor cells respond poorly to either therapy regimen. Our evidence that K17 correlates with PFS following treatment with gemcitabine- and 5-FU–based therapies indicates that K17 could also serve as a predictive biomarker to guide treatment intervention. Thus, to our knowledge, the current study is the first to define a threshold for positive K17 test results, as required to develop a clinically deployable biomarker for prognostic and predictive stratification.

Although K17 has emerged as a defining biomarker of the most aggressive forms of PDAC and is currently being tested in phase 2 clinical trials (NCT04469556, NCT02047474, and NCT03991962), the field has not established thresholds to classify patients as low or high risk based on K17 expression. Prior work from our group demonstrated that K17 expression in PDAC, measured by RNA sequencing or immunohistochemistry, is an independent negative prognostic biomarker that stratifies clinical outcomes for cases that are diagnosed by needle aspiration biopsy or resection.^4,15-18^ The current study built on this foundation to define prognostic and predictive thresholds for K17 expression based on IHC, to confirm that K17 expression correlates with clinicopathologic features in a retrospective cohort that included cases from a wide range of community-based, regional, and academically affiliated hospitals. While PDAC has a dismal OS, the ability to distinguish patients with the most aggressive forms of this disease vs those that are likely to have relatively extended survival could have an enormous impact on patients with PDAC as they adjust their health care management.^19^

To date, there is a lack of treatment-informing biomarkers in pancreatic cancer, with few exemptions like mismatch repair-deficient/microsatellite instability-high and homologous recombination deficiency status only used to select patients for checkpoint and poly(ADP-ribose) polymerase inhibitors. Although a wide range of other prognostic biomarkers have been reported, including CA 19-9, hENT1, SMAD4/DPC4, and hCNT1/hCNT3, none have yet been integrated into the routine diagnostic workup of PDAC.^20^ GATA6, a surrogate biomarker of the classical subtype, is associated with improved OS in patients with PDAC treated with modified FOLFIRINOX but not in patients treated with gemcitabine-based regimens,^21^ consistent with our current findings. However, this group did not propose that selection of a chemotherapeutic regimen should be based on biomarker expression. By contrast, we demonstrated that patients whose tumors express low K17 have better survival when treated with 5-FU–based therapy compared to those treated with gemcitabine-based regimens. Our observations would therefore have several potential clinical implications. For example, knowing which chemotherapy regimen is more effective in a subgroup of patients (low K17) could significantly improve patients’ clinical outcome.

Additionally, understanding that patients with high K17 respond equally poorly to both regimens could help direct the choice of a more tolerable chemotherapy like gemcitabine and thereby improve their quality of life. These findings also promote the study of chemosensitivity and resistance mechanisms that can help find more effective treatment choices for high K17-expressing patients who are unresponsive to current chemotherapy altogether. Prospective, randomized controlled clinical studies, however, need to be performed to determine if K17 test results could be used as a criterion to optimize therapeutic efficacy and to minimize the side effects attributable to agents that are unlikely to provide a survival advantage.

Limitations of this study are that each case was evaluated based on IHC of sections from a single, however most large, representative tissue block. Given the heterogeneous distribution of K17-expressing cells in many cases, the use of a single block, rather than performing IHC on every block of the PDACs, could have resulted in misclassification of overall levels of expression, dichotomized as high vs low for K17. This study was also limited to the scoring of K17 expression at the primary site (within the pancreas) and thus did not address patterns of expression in areas of invasion beyond the pancreas or in local or distant metastasis. Additionally, manual scoring of IHC results have the potential to introduce operator/reader variability. Thus, the development of an automatic IHC K17 detection by IHC could provide a further opportunity to standardize the deployment of a K17-based biomarker strategy in a clinical laboratory setting. Despite these limitations, K17 was highly prognostic for survival and predicted chemotherapeutic response, suggesting that the effects of K17 on biologic aggression, as summarized by Baraks et al,^22^ are profound. Prospective randomized controlled clinical studies are still indicated, however, to support the translation of this biomarker to clinical practice.

In conclusion, K17 expression has been validated as a robust prognostic biomarker and was significantly correlated with poor OS in 2 large, independent cohorts of patients with PDAC. Furthermore, our results indicate that K17 expression predicts PDAC resistance to gemcitabine-based chemotherapies in advanced-stage and/or LN-positive PDACs and helps identify a subgroup of patients who have enhanced therapeutic response to 5-FU–based therapies that may optimize therapeutic efficacy for patients with PDAC. These observations may guide further prospective studies to develop K17 as a prognostic and predictive biomarker in both adjuvant and palliative settings, providing an advancement in the selection of individualized treatment of patients with this devastating cancer. Additionally, incorporating K17 IHC testing as a predictive marker test will yield rapid results to inform the best chemotherapy based on the tumor’s expression profile.

## Supplementary Material

aqae038_suppl_Supplementary_Tables_1_Figures_1-5

## Notes

Funding: This work was supported by academic enrichment funds of the Department of Pathology at the Renaissance School of Medicine, Stony Brook University, the Pancreatic Cancer Action Network, and Perthera. K.R.S. and L.F.E.-H. received support from a Pancreatic Cancer Action Network Acceleration Network (18–65-SHRO). L.F.E.-H. also received awards from the National Cancer Institute (R01CA274355-01, DP2CA280625-01, R00 CA226342-01); the William Raveis Charitable Fund/Rachleff Innovator of the Damon Runyon Cancer Research Foundation; the AACR Career Development Award to Further Diversity, Equity, and Inclusion in Pancreatic Cancer Research; and the Dr. Ralph and Marian Falk Medical Research Trust, Bank of America, Private Bank.

Conflict of interest disclosure: K.R.S. and L.F.E.-H. are consultants for KDx Diagnostics. E.M.B. is an employee of Perthera and owns stocks in the company. E.F.P. has received compensation as an officer of Perthera and owns stock in the company. He also has consulted for Theralink Technologies and received compensation as chair of the Science Advisory Board and owns stock in the company. The additional authors have nothing to disclose.

Data-sharing statement: The data sets generated during and/or analyzed during the current study are not publicly available due to IRB regulations. All requests for data should be submitted to the corresponding authors for consideration.
