# Supplementary material for: Keratin 17 is a prognostic and predictive biomarker in pancreatic ductal adenocarcinoma
Source: Am J Clin Pathol. 2024 Apr 20;162(3):314–26. doi: 10.1093/ajcp/aqae038 (PMC11369068; doi:10.1093/ajcp/aqae038)
Supplement: aqae038_suppl_Supplementary_Tables_1_Figures_1-5 [file aqae038_suppl_supplementary_tables_1_figures_1-5.docx]

**Supplementary Figures**

**Supplementary Figure 1.**


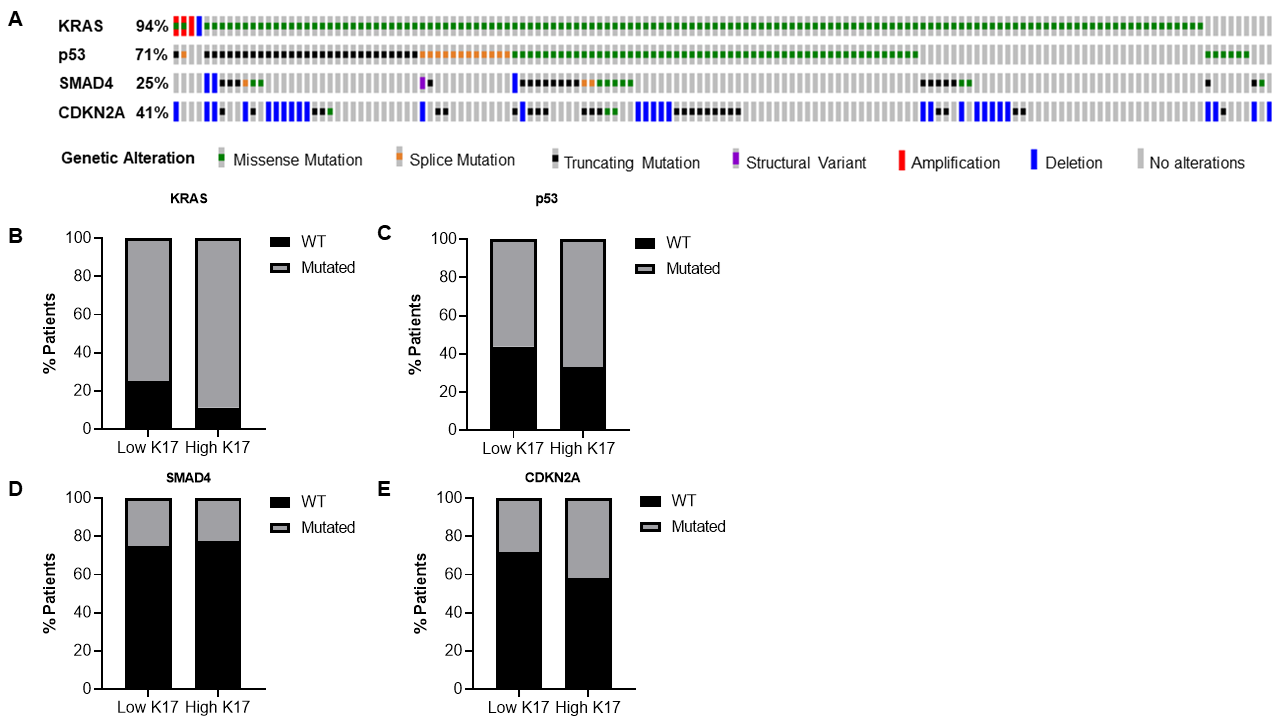


**Supplementary Figure 2.**

**Supplementary Figure 3.**

**Supplementary Figure 4.**

**Supplementary Figure 5.**

**Supplementary Table 1.**

|  | **K17 Score** | | | | |
| --- | --- | --- | --- | --- | --- |
| **Case** |  |  |  |  |  |
| **1** | **Block 1** | **Block 2** | **Block 3** | **Block 4** | **Block 5** |
| Pathologist A | 100 | 90 | 95 | 95 | 95 |
| Pathologist B | 100 | 95 | 90 | 90 | 95 |
| **2** | **Block 1** | **Block 2** | **Block 3** | **Block 4** | **Block 5** |
| Pathologist A | <1 | 5 | <1 | 0 | <1 |
| Pathologist B | <1 | 5 | 0 | 0 | 0 |
| **3** | **Block 1** | **Block 2** | **Block 3** | **Block 4** | **Block 5** |
| Pathologist A | 60 | 60 | 5 | 70 | 95 |
| Pathologist B | 70 | 50 | 10 | 30 | 90 |
| **4** | **Block 1** | **Block 2** | **Block 3** | **Block 4** | **Block 5** |
| Pathologist A | 5 | Tissue lost during processing | 10 | 20 | 80 |
| Pathologist B | 10 |  | 15 | 20 | 70 |
| **5** | **Block 1** | **Block 2** | **Block 3** | **Block 4** | **Block 5** |
| Pathologist A | 10 | 10 | 15 | 25 | 10 |
| Pathologist B | 5 | 5 | 15 | 20 | 15 |
| **6** | **Block 1** | **Block 2** | **Block 3** | **Block 4** | **Block 5** |
| Pathologist A | 90 | 90 | 85 | 90 | 70 |
| Pathologist B | 90 | 95 | 75 | 80 | 80 |
| **7** | **Block 1** | **Block 2** | **Block 3** | **Block 4** | **Block 5** |
| Pathologist A | 40 | 5 | 15 | 5 | 5 |
| Pathologist B | 30 | 10 | 15 | 10 | 5 |
| **8** | **Block 1** | **Block 2** | **Block 3** | **Block 4** | **Block 5** |
| Pathologist A | 40 | 90 | 5 | 20 | 30 |
| Pathologist B | 50 | 80 | 5 | 15 | 25 |
| **9** | **Block 1** | **Block 2** | **Block 3** | **Block 4** | **Block 5** |
| Pathologist A | 20 | 15 | 70 | 80 | 60 |
| Pathologist B | 25 | 10 | 95 | 75 | 75 |
| **10** | **Block 1** | **Block 2** | **Block 3** | **Block 4** | **Block 5** |
| Pathologist A | 80 | 70 | 40 | 25 | 70 |
| Pathologist B | 80 | 30 | 50 | 15 | 75 |
